# Admission Respiratory Rate-Oxygenation Index and Outcome of Acute Respiratory Illness in a Pediatric Intensive Care Unit

**DOI:** 10.7759/cureus.111005

**Published:** 2026-06-16

**Authors:** Vijendra Singh, Umesh Pandwar, Navindana, Manjusha Goel

**Affiliations:** 1 Paediatrics, Gandhi Medical College, Bhopal, Bhopal, IND

**Keywords:** acute respiratory illness, mortality predictor, pediatric intensive care, positive pressure ventilation, respiratory rate-oxygenative index, rox index

## Abstract

Background

Acute respiratory illness (ARI) remains a leading cause of pediatric intensive care unit (PICU) admissions and mortality in low- and middle-income countries. Early identification of children at risk of deterioration or ventilatory support is essential to improve outcomes. The Respiratory Rate-Oxygenation (ROX) Index, defined as (peripheral oxygen saturation (SpO₂)/fraction of inspired oxygen (FiO₂))/respiratory rate, is a non-invasive bedside tool validated in adults with respiratory failure; however, pediatric evidence is scarce.

Objectives

To evaluate the association of admission ROX Index with (1) mortality and (2) the requirement for positive-pressure ventilation (PPV) among children with ARI admitted to the PICU.

Methods

A prospective observational study was conducted among 200 consecutively admitted children aged two months to five years with ARI at a tertiary-care hospital. Demographic, anthropometric, and physiological parameters were recorded at admission. The ROX index was computed as (SpO₂/FiO₂)/respiratory rate. Primary outcomes were in-hospital mortality and need for PPV. Data were analyzed using non-parametric tests and receiver operating characteristic (ROC) analysis; *p*<0.05 was considered significant (STATA v14; StataCorp LLC, College Station, TX, US).

Results

The median age was seven months (IQR 4-12); 131 (65.5%) were male patients, and severe undernutrition (weight-for-age Z-score <−3 SD) was observed in 36 (18%) children. Overall mortality was 7% (14/200). The median admission ROX index was lower among non-survivors (5.6 (5.2-6.4)) than survivors (6.3 (5.9-7.1); *p*=0.002). The ROX index showed moderate discrimination for mortality (AUC=0.746; 95% CI 0.62-0.87) with an optimal cut-off ≤6.4 (sensitivity 100%, specificity 46%). For predicting PPV requirement, discrimination was good (AUC = 0.867; 95 % CI 0.80-0.93) with a cut-off ≤6.2 (sensitivity 88%, specificity 82%). Lower ROX values correlated with higher FiO₂ needs, greater tachypnea, and longer PICU stay (median eight days (IQR 6-11)).

Conclusion

The admission ROX index is a simple, non-invasive, and reliable predictor of mortality and ventilatory support requirement in children with acute respiratory illness. A cut-off ≤6.2 showed good discriminative accuracy for identifying high-risk cases. Its integration into triage protocols can strengthen early recognition and management of severe pediatric ARI in resource-limited PICUs.

## Introduction

Acute respiratory illnesses (ARIs) remain one of the foremost causes of morbidity and mortality among children worldwide, particularly in low- and middle-income countries [1]. Based on the Global Burden of Disease 2019 estimates, lower respiratory infections (including pneumonia and bronchiolitis) remain a major contributor to under-five mortality. In 2019, there were approximately 2.4 million deaths globally attributable to lower respiratory infections, and in children under five, these infections continue to be among the leading causes of death [2]. In India and other resource-constrained settings, ARIs continue to dominate pediatric intensive care unit (PICU) admissions, especially during seasonal surges of viral epidemics. The high burden of respiratory distress in infancy and early childhood places enormous strain on critical care resources, highlighting the urgent need for rapid and reliable bedside tools for early risk assessment [3].

Timely identification of children at risk of respiratory deterioration is a cornerstone of pediatric critical care. Although several prognostic models, such as the Pediatric Risk of Mortality (PRISM) [4] and Pediatric Index of Mortality (PIM) scores [5], are available, their use in routine care is often limited by the requirement for multiple laboratory parameters and complex calculations. These tools, though robust, may not be feasible for continuous, real-time decision-making in busy or resource-limited environments. Clinicians frequently depend on subjective assessment and evolving clinical judgment to determine when escalation to non-invasive or invasive ventilation is warranted; an approach vulnerable to variability and delay [6].

The respiratory rate-oxygenation (ROX) index, calculated using peripheral oxygen saturation (SpO₂), fraction of inspired oxygen (FiO₂), and respiratory rate (RR), integrates two fundamental physiological parameters like oxygenation and ventilatory effort into a single, non-invasive metric [7]. Originally developed to predict high-flow nasal cannula therapy success in adults with acute hypoxemic respiratory failure, the ROX index has shown promise as a simple, continuous predictor of disease severity. However, evidence in pediatric populations remains limited, and optimal threshold values have not been clearly defined across age groups or disease spectrums.

Given the paucity of data in critically ill children, especially in Indian PICU settings, this study aimed to evaluate the association of admission ROX index values with mortality and the need for positive-pressure ventilation in children with ARI. Establishing the prognostic utility of this readily obtainable index could significantly strengthen early triage and clinical decision-making in pediatric critical care.

## Materials and methods

This was a prospective observational study conducted in the PICU of the Department of Pediatrics, a tertiary-care referral centre in Bhopal. The study was conducted over 18 months, from June 2024 to September 2025, encompassing both the pre- and post-monsoon seasons to capture the peak incidence of respiratory illnesses. All children aged two months to five years admitted with a clinical diagnosis of ARI were eligible for inclusion. ARI was operationally defined as the presence of acute respiratory distress (tachypnea, retractions, nasal flaring, or grunting) with or without hypoxia (SpO₂<92% on room air). Children with known chronic cardiopulmonary diseases (such as congenital heart disease, bronchopulmonary dysplasia, cystic fibrosis), immunodeficiency disorders, neuromuscular diseases, or other chronic systemic illnesses) were excluded to avoid confounding due to pre-existing respiratory compromise.

The sample size was estimated using the standard single-proportion formula: z²pq/d², where Z=1.96 (for 95% confidence), p=estimated prevalence of ARI among PICU admissions is 15% (as per the hospital statistics for the year 2023), q=1 − p=85%, and d=allowable error of 5%. Substituting these values yielded n≈196, which was rounded to a final sample of 200 children to account for potential attrition or incomplete data. A consecutive sampling approach was adopted. All eligible children who met the inclusion criteria during the study period were enrolled consecutively at the time of PICU admission until the target sample size was achieved. This method minimized selection bias and ensured representation of the routine clinical case-mix of pediatric respiratory illnesses.

Data were collected using a pre-tested, semi-structured questionnaire developed by the investigators after an extensive literature review and consultation with senior pediatric intensivists. The tool captured sociodemographic variables (age, sex, residence, parental education), anthropometric indices (weight-for-age Z-score classified as per WHO standards), and physiological parameters including SpO₂, FiO₂, RR, heart rate, and temperature at admission.

The ROX index was calculated using the formula:

ROX index = (SpO₂/FiO₂) / RR

Outcome variables included (1) the need for positive-pressure ventilation (PPV), defined as initiation of non-invasive ventilation (continuous positive airway pressure (CPAP) or bilevel positive airway pressure (BiPAP) or invasive mechanical ventilation, and (2) in-hospital mortality. The questionnaire was pre-tested on ten children admitted with ARI (excluded from the final cohort) to assess clarity, content relevance, and feasibility. Based on feedback, minor modifications were made to optimize terminology and uniformity of data recording. Content validity was reviewed and approved by a three-member expert panel comprising two senior pediatricians and one biostatistician.

At admission, the attending pediatric resident recorded all baseline variables prior to initiation of supplemental oxygen. SpO₂ was measured using a motion-artifact-free calibrated pulse oximeter (Mindray PM-60, Shenzhen Mindray Bio-Medical Electronics Co., Ltd., Shenzhen, Guangdon) applied to the right hand in ambient air or at the lowest FiO₂ required for clinical stability. FiO₂ was estimated according to the oxygen delivery device used, following standard pediatric oxygen-delivery conversion charts (room air=0.21; nasal prongs=0.24-0.28; CPAP=0.30-0.40; invasive ventilation>0.40). RR was counted manually for one full minute during quiet breathing using standard pediatric assessment techniques. All measurements were repeated 30 minutes after initiation of respiratory support to obtain post-oxygenation parameters.

Children were followed throughout their PICU stay until discharge or death. For those requiring PPV, the indication, mode, and duration of support were documented. Each day, the principal investigator cross-verified the case sheets for completeness and internal consistency. Prior to data collection, the pediatric resident underwent two structured training sessions on accurate measurement of respiratory parameters, FiO₂ estimation, operational definitions, and standardized documentation procedures. A competency checklist was maintained to ensure adherence. Inter-observer variability was minimized through periodic supervision and duplicate readings on randomly selected cases. Pulse oximeters, flow meters, and oxygen delivery systems were calibrated weekly under supervision of the biomedical engineering unit. Random cross-checks of 10% of entries were performed daily by the principal investigator to verify accuracy, and outliers were re-examined against source records before final data entry.

Data were entered into Microsoft Excel 2019 (Microsoft Corp., Redmond, WA, USA) and analyzed using STATA version 14.2 (StataCorp LLC, College Station, TX, US). Continuous variables were summarized as mean ± standard deviation (SD) or median (interquartile range or IQR) depending on distribution assessed by the Shapiro-Wilk test. Categorical variables were expressed as frequencies and percentages. The association between ROX Index and mortality was examined using the Mann-Whitney U test, while categorical comparisons were analyzed using the Chi-square or Fisher’s exact test as appropriate. Predictive validity was assessed using receiver operating characteristic (ROC) curve analysis, with area under the curve (AUC), sensitivity, specificity, and Youden J statistics reported. A two-tailed p<0.05 was considered statistically significant.

Approval was obtained from the Institutional Ethics Committee (Ref No: GMCB/IEC/2022/214) prior to data collection. Written informed consent was secured from parents or legal guardians after explaining study objectives, procedures, potential risks, and the voluntary nature of participation. Confidentiality was maintained through anonymized codes, and data were used solely for academic purposes.

## Results

A total of 200 children were enrolled. The median age was seven months, and 131 (65.5%) were male patients. As shown in Table 1, 91 (45.5%) had normal nutritional status, 73 (36.5%) were moderately undernourished, and 36 (18%) were severely undernourished.

**Table 1:** Baseline demographic and clinical characteristics of the study population (N=200) Data are presented as n (%) or median (interquartile range or IQR) as appropriate. ROX index was calculated as (SpO₂/FiO₂)/respiratory rate (RR). SpO₂, peripheral oxygen saturation; FiO₂, fraction of inspired oxygen; ROX index, respiratory rate-oxygenation index; PICU, Pediatric Intensive Care Unit; IQR, interquartile range.

Characteristic	Category / Statistic	Value
Age (months)	Median (IQR)	7 (4-12)
Gender	Male	131 (65.5%)
	Female	69 (34.5%)
Nutritional status (Z-score)	> –2 SD	91 (45.5%)
	–2 to –3 SD	73 (36.5%)
	< –3 SD	36 (18.0%)
SpO₂ at admission (%)	Median (IQR)	88 (86-89)
FiO₂ at admission	Median (IQR)	0.21 (0.18-0.26)
Respiratory rate (/ min)	Median (IQR)	66 (60-72)
Admission ROX Index (SpO₂ / FiO₂ / RR)	Median (IQR)	6.3 (5.8-7.0)
Length of PICU stay (days)	Median (IQR)	5 (4-6)
Outcome	Discharged	186 (93.0%)
	Died	14 (7.0%)

The median admission SpO₂ was 88 (86-89%) and RR 66 (60-72) breaths per minute. The corresponding median ROX index was 6.3 (IQR 5.8-7.0). The median PICU stay was five (four to six) days, and overall mortality was 14 (7%).

As shown in Table 2, 14 (7%) children died during the PICU stay.

**Table 2:** Comparison of baseline and physiological variables by outcome (mortality) among children with acute respiratory illness in PICU (N=200) Data are presented as n (%) or median (IQR). Continuous variables were compared using the Mann–Whitney U test, while categorical variables were analysed using Chi-square or Fisher’s exact test as appropriate. ^†^Post-oxygen ROX values were calculated after initiation of oxygen therapy or respiratory support. ROX index, (SpO₂/FiO₂)/RR; SpO₂, peripheral oxygen saturation; FiO₂, fraction of inspired oxygen; ROX index, respiratory rate-oxygenation index; FiO₂, fraction of inspired oxygen; RR, respiratory rate; CPAP, continuous positive airway pressure; IQR, interquartile range.

Variable	Survived (n=186)	Died (n=14)	Test statistic	p-value
Age (months)	7 (4-12)	8 (5-18)	Mann–Whitney U=1168	0.62
Gender			χ² = 1.12	0.29
Male	120 (64.5%)	11 (78.6%)		
Female	66 (35.5%)	3 (21.4%)		
Nutritional status (Z-score)			Fisher’s exact	0.001
>−2 SD (normal)	110 (59.1%)	5 (35.7%)		
−2 to −3 SD (moderate)	47 (25.3%)	1 (7.1%)		
<−3 SD (severe)	29 (15.6%)	8 (57.1%)		
SpO₂ (%) at admission	88 (86-89)	84 (82-86)	Mann–Whitney U = 758	<0.001
FiO₂ (fraction)	0.21 (0.21-0.28)	0.30 (0.21-0.40)	Mann–Whitney U = 932	0.014
Respiratory rate (/ min)	65 (60- 72)	72 (68-76)	Mann–Whitney U = 801	0.003
ROX Index (SpO₂ / FiO₂ / RR)	6.3 (5.9-7.07)	5.6 (5.2-6.4)	Mann–Whitney U = 890	0.002
Post-oxygen ROX (Nasal Prongs)^†^	9.6 (8.5-10.8)	7.2 (6.4-8.1)	Mann–Whitney U = 744	<0.001
Post-oxygen ROX (Ventilation / CPAP)^†^	7.9 (7.3-8.5)	6.1 (5.5-7.2)	Mann–Whitney U = 788	0.002
Length of stay (days)	5 (4-6)	8 (6-11)	Mann–Whitney U = 874	0.008

Age and sex distributions were comparable between survivors and non-survivors. Severe undernutrition (<-3 SD) was significantly more frequent among non-survivors (p=0.001). Non-survivors presented with lower admission SpO₂, higher FiO₂ requirements, and higher RRs (all p<0.05). The admission ROX index was significantly lower in non-survivors (median 5.6 (IQR 5.2-6.4)) compared with survivors (6.3 (5.9-7.1); p=0.002). Post-oxygen ROX values remained consistently lower among non-survivors across both nasal-prong and CPAP subgroups, and the median PICU stay was significantly longer in non-survivors (p=0.008).

**Figure 1:**
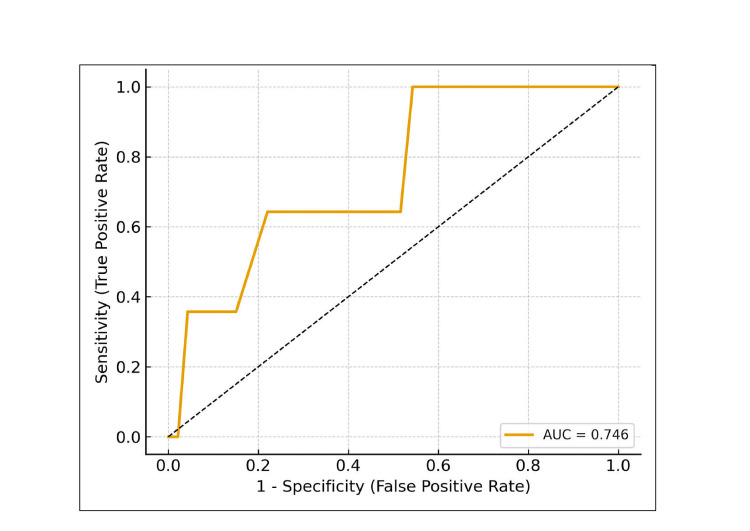
ROC for admission ROX index predicting mortality ROC, receiver operating characteristic; ROX index, (SpO₂/FiO₂)/RR; SpO₂, peripheral oxygen saturation; FiO₂, fraction of inspired oxygen; ROX index, respiratory rate-oxygenation index.

**Figure 2:**
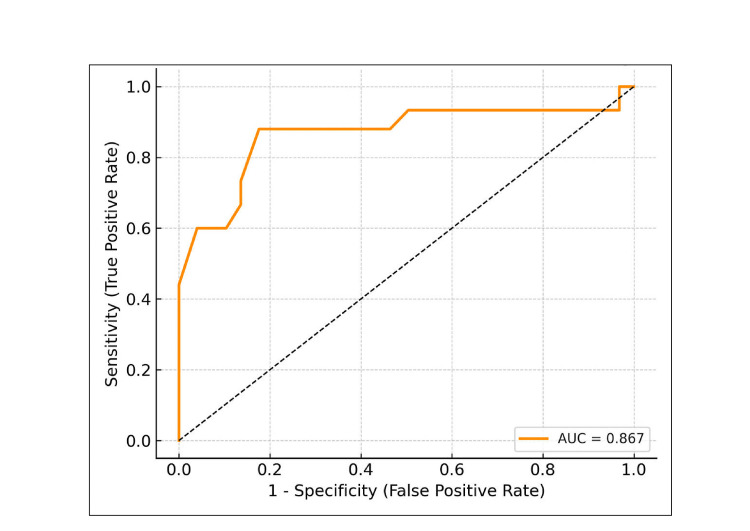
ROC for admission ROX index predicting PPV requirement ROC, receiver operating characteristic; PPV, positive-pressure ventilation; ROX index, (SpO₂/FiO₂)/RR; SpO₂, peripheral oxygen saturation; FiO₂, fraction of inspired oxygen; ROX index, respiratory rate-oxygenation index.

The receiver operating characteristic (ROC) analysis showed that the admission ROX index had a moderate ability to predict mortality and a good ability to predict the need for PPV (Table 3, Figures 1, 2).

**Table 3:** ROC analysis of admission ROX index for predicting mortality and requirement for PPV among children with acute respiratory illness in the PICU (N=200) ROC, receiver operating characteristic; AUC, area under the curve; CI, confidence interval; PPV, positive-pressure ventilation; ROX index, (SpO₂/FiO₂)/RR; SpO₂, peripheral oxygen saturation; FiO₂, fraction of inspired oxygen; ROX index, respiratory rate-oxygenation index; RR, respiratory rate. Optimal cut-off values were determined using the Youden Index. *Statistically significant (p<0.05). Lower ROX index values indicate poorer oxygenation and increased risk of adverse clinical outcomes.

Parameter	Mortality prediction	PPV requirement prediction
AUC (95% CI)	0.746 (0.62-0.87)	0.867 (0.80-0.93)
Interpretation	Moderate discriminative ability	Good discriminative ability
Optimal cut-off (Youden Index)	≤6.4	≤6.2
Sensitivity (%)	100	88
Specificity (%)	46	82
Positive predictive value (%)	20.6	76.3
Negative predictive value (%)	100	86.9
Youden J statistic	0.457	0.704
p-value (AUC > 0.5)	<0.001*	<0.001*

For mortality, the AUC was 0.746 (95% CI 0.62-0.87; p<0.001), indicating moderate discriminative performance between survivors and non-survivors. The optimal admission ROX cut-off of ≤6.4 provided 100% sensitivity and 46% specificity, with a negative predictive value of 100%. For predicting PPV requirement, the AUC was 0.867 (95% CI 0.80-0.93; p<0.001), denoting good discrimination. A threshold of ≤6.2 achieved 88% sensitivity and 82% specificity, effectively identifying children at higher risk of requiring ventilatory support. Overall, 75 (37.5%) children required PPV during their PICU stay.

## Discussion

In this prospective study of children admitted with ARI, the admission ROX index demonstrated moderate accuracy for predicting mortality and good discriminative ability for identifying the need for PPV. Lower ROX index scores were significantly associated with adverse outcomes, with optimal cut-offs of ≤6.4 for mortality (AUC=0.746) and ≤6.2 for ventilatory support (AUC=0.867), effectively identifying high-risk patients. Children with lower admission ROX indices were more likely to be malnourished, require higher FiO₂ concentrations, and experience longer PICU stays, reflecting greater physiological severity. These findings underscore the potential utility of the ROX Index as a simple, bedside triage tool for early risk stratification in resource-limited pediatric intensive care settings. Notably, this study provides one of the first prospective validations of the admission ROX index in a pediatric cohort, thereby extending evidence from predominantly adult populations to children with ARI.

Comparison with existing literature

In a Korean study by Choi et al. (2023) [8], conducted among 107 children receiving high-flow nasal cannula (HFNC) therapy, ROX values between 6.88 and 10.16 effectively distinguished successful weaning from HFNC failure. Similarly, Kannikeswaran et al. (2022) [9] examined infants with bronchiolitis and found that children in the lowest ROX quartile (<5.39) were three times more likely to require PPV compared to those with higher values and reported a threshold of ≤8.5 for PPV prediction, whereas in our cohort the admission ROX cut-off was ≤6.2. These results closely mirror our threshold for PPV prediction, although their higher age-specific RR range and inclusion of predominantly viral bronchiolitis cases may explain the slight difference in cut-off estimates. Furthermore, their study measured ROX at HFNC initiation rather than at PICU admission, while our study evaluated baseline ROX values before oxygen supplementation, which might better reflect intrinsic disease severity rather than therapeutic response.

The findings of Calderón-Salavarría and Barreiro-Casanova (2024) [10] and Yıldızdaş et al. (2020) [11] also support the dynamic use of the ROX index. Both demonstrated that serial ROX measurements during HFNC therapy could predict treatment failure with good discriminative ability (AUC 0.7-0.8). Our use of a single admission ROX value achieved a comparable AUC (0.87), suggesting that even a point measurement can provide strong prognostic information, especially in resource-limited settings where repeated measurements may be impractical. The congruence across these studies reinforces the biological plausibility of ROX as a marker of disease severity, integrating oxygenation efficiency (SpO₂/FiO₂) and ventilatory effort (RR), both of which deteriorate as respiratory failure progresses.

Adult data further validate these associations. In community-acquired pneumonia, Reyes et al. (2022) [12] found ROX <14.8 predictive of invasive mechanical ventilation (AUC 0.77), while Nishiyama et al. (2023) [13] demonstrated that higher ROX tertiles correlated with improved survival in acute respiratory distress syndrome (ARDS). Also, Liu et al. (2024) [14] reported an L-shaped relationship between ROX and mortality, with steep risk increases below 8.3. The lower thresholds observed in pediatric studies, including ours, can be explained by age-related physiological differences. Higher baseline RRs and smaller tidal volumes in children result in naturally lower ROX values even at comparable oxygenation levels.

Clinical implications

The findings of this study have important clinical implications for pediatric critical care, particularly in low- and middle-income countries where diagnostic and monitoring resources are limited. The ROX index, derived from simple bedside parameters like oxygen saturation, FiO_2_, and RR offers a rapid, objective tool to identify children at risk of respiratory failure at the time of admission. Incorporating this index into routine triage could facilitate early recognition of children likely to require ventilatory support, enabling timely escalation of care and more efficient allocation of scarce critical care resources [15]. The high negative predictive value observed in our study (98.8%) suggests that children with higher ROX scores can be safely managed with non-invasive support and close observation, thereby reducing unnecessary intubation and ventilator use. Additionally, since the ROX index can be calculated using data readily available in emergency departments or general wards, it can serve as an early warning indicator before PICU transfer, supporting the development of rapid response protocols.

Strengths and limitations

A major strength of this study is its prospective design, which minimizes recall and selection biases commonly seen in retrospective analyses. Consecutive patient enrollment, standardized measurement of physiological parameters, and the use of validated statistical tools (ROC analysis and Youden index) add robustness to the findings. Unlike most previous pediatric studies that focused solely on high-flow oxygen cohorts, our study examined the ROX index across all forms of ARI, making the results more generalizable to typical PICU populations. However, several limitations should be acknowledged. Being a single-center study, external validity may be limited, and local disease epidemiology could influence results. Serial ROX measurements were not assessed, which might have provided additional prognostic insights. Moreover, children with chronic pulmonary or cardiac comorbidities were excluded to avoid confounding, but this may restrict applicability to those high-risk groups. Future multicentric validation is needed to refine cut-offs across age and disease subtypes.

## Conclusions

The ROX index is a simple, non-invasive, and readily available bedside tool for assessing children with ARI admitted to the PICU. Lower admission ROX index values were associated with an increased need for PPV and poorer clinical outcomes. The index demonstrated potential utility in early risk stratification and identification of children at risk of respiratory deterioration. Its use may facilitate timely escalation of respiratory support and improve clinical decision-making in resource-limited settings. Given its ease of calculation and reliance on routinely measured parameters, the ROX index can be incorporated into routine pediatric critical care assessment. Further multicenter studies are warranted to validate optimal pediatric cut-off values and establish the role of ROX index in standardized management protocols.
